# Ocular Physiologically Based Pharmacokinetic Modeling for Ointment Formulations

**DOI:** 10.1007/s11095-020-02965-y

**Published:** 2020-11-19

**Authors:** Maxime Le Merdy, Jessica Spires, Viera Lukacova, Ming-Liang Tan, Andrew Babiskin, Xiaoming Xu, Liang Zhao, Michael B. Bolger

**Affiliations:** 1 Simulations Plus, Inc., 42505 10th Street West, Lancaster, California 93534 USA; 2 Food and Drug Administration, CDER/OGD/ORS/DQMM, 10903 New Hampshire Avenue, Silver Spring, Maryland 20993 USA; 3 Food and Drug Administration, CDER/OPQ/OTR/DPQR, 10903 New Hampshire Avenue, Silver Spring, Maryland 20993 USA

**Keywords:** ocular PBPK, ophthalmic ointment, PBPK, product development

## Abstract

**Purpose:**

The purpose of this study is to show how the Ocular Compartmental Absorption & Transit (OCAT™) model in GastroPlus^®^ can be used to characterize ocular drug pharmacokinetic performance in rabbits for ointment formulations.

**Methods:**

A newly OCAT™ model developed for fluorometholone, as well as a previously verified model for dexamethasone, were used to characterize the aqueous humor (AH) concentration following the administration of multiple ointment formulations to rabbit. The model uses the following parameters: application surface area (SA), a fitted application time, and the fitted Higuchi release constant to characterize the rate of passage of the active pharmaceutical ingredient from the ointment formulations into the tears in vivo.

**Results:**

Parameter sensitivity analysis was performed to understand the impact of ointment formulation changes on ocular exposure. While application time was found to have a significant impact on the time of maximal concentration in AH, both the application SA and the Higuchi release constant significantly influenced both the maximum concentration and the ocular exposure.

**Conclusions:**

This initial model for ointment ophthalmic formulations is a first step to better understand the interplay between physiological factors and ophthalmic formulation physicochemical properties and their impact on in vivo ocular drug pharmacokinetic performance in rabbits.

**Supplementary Information:**

The online version contains supplementary material available at 10.1007/s11095-020-02965-y.

## Introduction

The United States (US) Food and Drug Administration (FDA) may recommend in vivo and/or in vitro testing to establish the bioequivalence (BE) of ocular drug products with complex dosage forms. The selection of the method depends upon the most current understanding of FDA in relation to the information collected by the study, the analytical methods available, and the nature of the drug product. In general, BE testing should use the most accurate, sensitive, and reproducible approach that is able to capture differences between the test and reference products in terms of drug concentrations at the site of action (1). Different approaches, such as a combination of in vitro characterization studies, aqueous humor (AH) pharmacokinetic (PK) BE studies and/or comparative clinical endpoint BE studies have been recommended to demonstrate BE for ophthalmic products depending on the drug product’s active pharmaceutical ingredient (API), dosage form, indication, mechanism of action, and scientific understanding of drug release and disposition in the eye (2). However, these in vivo clinical studies have inherent study design and sampling challenges that may limit the development of ophthalmic generic drugs (3). For complex (i.e., non-solution) ophthalmic dosage forms, such as ointments, establishing BE without an in vivo study may be permissible if, in addition to the compositional similarity, the generic formulation demonstrates similar physicochemical properties to the RLD (4); otherwise, additional evidence of BE such as an in vivo study would be recommended. Provided that about half of the currently marketed innovator ophthalmic drugs have at least one approved generic competitor and most are ophthalmic solutions (5), further research into the role of formulation physicochemical properties on ocular bioavailability is warranted and can be used to support product development and alternative BE approaches.

Ointment formulations represents approximately 8% of the ophthalmic products in the US (6). Because these formulations offer unique advantages over ophthalmic solutions such as increased pre-corneal residence time (7), they are used for localized treatment of eye diseases, such as glaucoma, infections and inflammatory conditions (8–10). The interactions between ointments physicochemical properties and complex ocular physiological factors on the clinical performance of the product is not well understood. Recent studies have evaluated how formulation and process factors influence product quality and in vitro performance of ophthalmic ointments (6,11,12). However, there is currently a limited understanding of how variability in the in vitro properties of an ophthalmic ointment product correlate to changes in therapeutic performance and more research would improve the development of ophthalmic ointment formulations for both new and generic drugs.

Physiologically based pharmacokinetic models (PBPK) were first introduced in the 1970s to support drug development from preclinical to clinical trials as they can reduce the cost and attrition rate in drug development (13). PBPK modeling provides insight into drug partitioning in different eye tissues that are usually not accessible and/or are challenging to sample in humans. This approach presents the unique advantage of combining formulation characteristics, that could be obtained in vitro, and the current best understanding of the ocular structure and physiology in the same platform. Ocular PBPK models are an important tool for biopharmaceutic formulation development (14,15).

The first physiologically based model describing ocular concentration-time profiles for different tissues in rabbits was developed in the late 1970s (16,17). PBPK models focusing on suspensions, (18,19), tear fluid pH variation in the pre-cornea (20), intravitreal administration of small molecular weight compounds and macromolecules (21–24), impact of melanin binding on ocular drug delivery (25), relationship between aqueous humor and plasma exposure (26), and description of the tear space for bioavailability calculations (27) were developed over the years. However, most of these studies focused on drug distribution within only a limited number of ocular tissues and addressed only limited aspects of formulation behavior. An ocular PBPK and mechanistic absorption model was recently developed and validated for multiple formulations and characteristics of ophthalmic suspensions in rabbit, such as particle size, viscosity, and dose (28). To our knowledge, a mathematical PBPK model has not been developed for ointment formulation yet.

The purpose of this article is to demonstrate the application of ocular PBPK and mechanistic absorption model to predict the impact of ophthalmic ointment characteristics on drug disposition in rabbit eyes using Dexamethasone (Dex) and Fluorometholone (Flm) as the model drugs.

This study includes: 1) the verification of the developed mechanistic ocular absorption model (OCAT™) in rabbits using concentration-time profiles of Flm in different ocular tissues and plasma for multiple formulations with different product characteristics; and 2) the investigation of the impact of dose and administered volume on drug disposition in rabbit eyes for ointment formulations.

## Materials and Methods

GastroPlus^®^ (version 9.8 Simulation Plus Inc., Lancaster, CA, USA) was used for computer simulation of Flm and Dex biodistribution in rabbit ocular tissues. The Ocular Compartmental Absorption & Transit (OCAT™) model describing ocular drug absorption and disposition details is presented in supplementary materials 1.

### Ointment Model Structure

For this project, a new ointment dosage form was created within GastroPlus^®^ to describe the observed data in rabbit for both case studies. The ointment formulation is treated as a controlled release formulation with the release of API described by the Higuchi equation (29,30) (E1):

E1 $$ \frac{d{m}_{free}}{dt}= SA\times {k}_{Higuchi}\times {t}^{-1/2} $$

where SA is the area of application on the eye, m_free_ is the amount released from the ointment and k_Higuchi_ is a fitted value of the Higuchi rate constant. The ointment is eliminated from the surface of the eyes through a zero-order process. Because the amount of ointment retained in the eye is believed to decrease due to blinking, it is assumed that the eliminated API enters the stomach through nasolacrimal drainage, but it is eliminated at a much slower rate than solutions and small particle size suspensions. The following equation describes the elimination of the ointment material from the ocular surface:

E2 $$ \frac{d{m}_{unrel}}{dt}=-{k}_{ointment} $$

where m_unrel_ is the unreleased amount of API remaining in the ointment and k_ointment_ is the zero-order rate constant describing the disappearance of the ointment material. k_ointment_ is automatically calculated by the software based on application time, a user-defined parameter:

E3 $$ {k}_{ointment}=\frac{dose}{application\ time} $$

Therefore, three parameters influence the behavior of this ointment formulation: the application SA, the Higuchi rate constant, and the application time. These parameters must be defined by the user in GastroPlus^®^.

### Flm Case Study

Model structure integrates an OCAT™ model (see supplementary materials 1 for details), describing ocular drug absorption and disposition, an Advanced Compartmental Absorption & Transit (ACAT™) model to capture intestinal absorption of Flm after being ingested through nasolacrimal drainage, and a mammalian one-compartment systemic distribution and clearance model. The clearance and volume of distribution were calculated based on the Flm molecular structure using ADMET Predictor® (version 9.5, Simulation Plus Inc., Lancaster, CA, USA.). Input parameters for Flm (Table I) were obtained from literature or were fitted to in vivo data.

**Table I:** Summary Of Parameter Values Implemented In The OCAT^™^-PBPK Model For Flm

Parameter		Definition	Value	Units
Flm Physicochemical properties				
	MWt	molecular weight	376.47^a,d^	g/mol
	logP(neutral)	Log Octanol/Water Partition Coefficient	2^b,d^	-
	Fu	Plasma unbound percent	19.1^a^	%
	B/P	Blood to Plasma concentration ratio	0.91^a^	
	Solubility (pH 7)	Maximum amount of Flm dissolved in water	0.0155^c^	mg/mL
Central compartment parameters				
	CL	Systemic clearance	0.44^a^	L/hr
	V_c_	Volume of central compartment	2.58^a^	L/kg
In Vitro measurement				
	Perm_Cornea vitro_	Cornea permeability in vitro	1.66 ^d^	x10^-5^ cm/s
OCAT^™^ parameters				
	Perm_Cornea_epi_	Cornea epithelium permeability	1.0^**^	x10^-5^ cm/s
	Perm_Cornea_str_	Cornea stroma permeability	0.80^**^	x10^-5^ cm/s
	Perm_Conjunctiva_	Conjunctiva permeability	1.52^a^	x10^-6^ cm/s
	Perm_AH._	AH permeability	1.5^**^	x10^-5^ cm/s
	Perm_ICB_	Iris-Ciliary Body permeability	7.75^a^	x10^-4^ cm/s
	Perm_Sclera_	Sclera permeability	2.85^a^	x10^-5^ cm/s
	Perm_Choroid_	Choroid permeability	5.10^a^	x10^-4^ cm/s
	Perm_Retina_	Retina permeability	1.74^a^	x10^-5^ cm/s
	Perm_V.H._	Vitreous Humor permeability	6.6^a^	x10^-6^ cm/s
	SAR_Choroid_	Choroid systemic absorption rate	7.64^a^	x10^-4^ s^-1^
	SAR_Retina_	Retina systemic absorption rate	1.2^a^	x10^-3^ s^-1^
	SAR_Conjunctiva_	Conjunctiva systemic absorption rate	1.32^a^	x10^-3^ s^-1^
	SAR_ICB_	ICB systemic absorption rate	1.01^a^	x10^-3^ s^-1^
	TF	Tear Flow rate	1.12^e^	μL/min
	DR	Drainage rate	1^e^	min^-1^
	V_pc_max_	Maximum pre-corneal volume	35^e^	μL
Ointment parameters				
	ASA	Application surface area	1.763^e^	cm^2^
	AT_50_	Application time for 50 μg dose	5^**^	h
	AT_25_	Application time for 25 μg dose	3.5^**^	h
	k_Higuchi_	Higuchi release constant	2.7^**^	x10^-6^ mg/(s^1/2^ cm^2^)

^a^ estimated using ADMET Predictor^®^ v9.5 or GastroPlus^®^ 9.8

^b^ (31)

^c^ (18)

^d^ (32)

^e^ default value in GastroPlus^®^ 9.8

** optimized parameters

The following assumptions were made for the OCAT™ model: 1) the administered dose is lost from the eye by overflow based on the maximum pre-corneal volume and the administered dose volume; 2) the particles of Flm suspension do not trigger excessive lacrimation; and 3) the drug is released from the ointment formulation into the tears where it can either be absorbed within both cornea and conjunctiva or be washed out the surface of the eye through drainage.

The initial model was developed against cornea and AH concentration time course data following the administration of a saturated solution of Flm (50 μL of Flm solution 4.0E-5 M) to rabbit (33). In vitro cornea permeability (1.66E-5 cm/s) (32) was first tested to describe the observed data. However, the cornea exposure was over predicted, therefore for the final model cornea epithelial (1.0E-5 cm/s), stromal (0.8E-5 cm/s) and aqueous humor (AH) (1.5E-5 cm/s) permeability were optimized in GastroPlus^®^. Two verification steps were performed using published AH concentration time course for suspensions, with differences in dose and particle size (PS): three suspensions (0.01%, 0.05% and 0.1%) with a mean PS of 2 μm for verification #1; and two suspensions (0.1% and 0.4%) with a mean PS of 10.4 μm and 6 μm, respectively, for verification #2 (18,34). For suspension formulations, a constant PS for all suspended material was assumed and based on the solubility, the mass of drug in solution and in suspended particles was calculated and administered using the Mixed Multiple Dose feature of GastroPlus^®^. The Lu, Frisela, and Johnson dissolution model was used to describe the dissolution of Flm suspended particles (35). Once the model was verified for multiple suspensions, it was used to simulate ointment formulations. AH concentration time courses following the administration of two similar 0.1% ointments (25 and 50 μL) were extracted from literature (34). Ointment application SA was assumed to be equal to the total cornea surface area, due to blinking that spread the formulation across the eye. The Higuchi release constant and application time were manually optimized to describe the observed AH data.

### Dex Case Study

An ocular model of Dex was previously developed and verified (28). For this case study, all model parameters were unchanged. Dex AH concentrations following the administration of a 0.1% ointment were extracted from literature (volume = 30 μL) (36). Ointment application SA was assumed to be equal to the total cornea surface area. The Higuchi release constant and the time of contact were optimized to describe the observed AH data.

### Parameter Sensitivity Analysis

The impact of application time, application SA, and Higuchi rate constant on Flm AH concentration time course in rabbit were assessed using parameter sensitivity analysis (PSA) in GastroPlus^®^.

## Results

### Flm Case Study

**Fig. 1:**
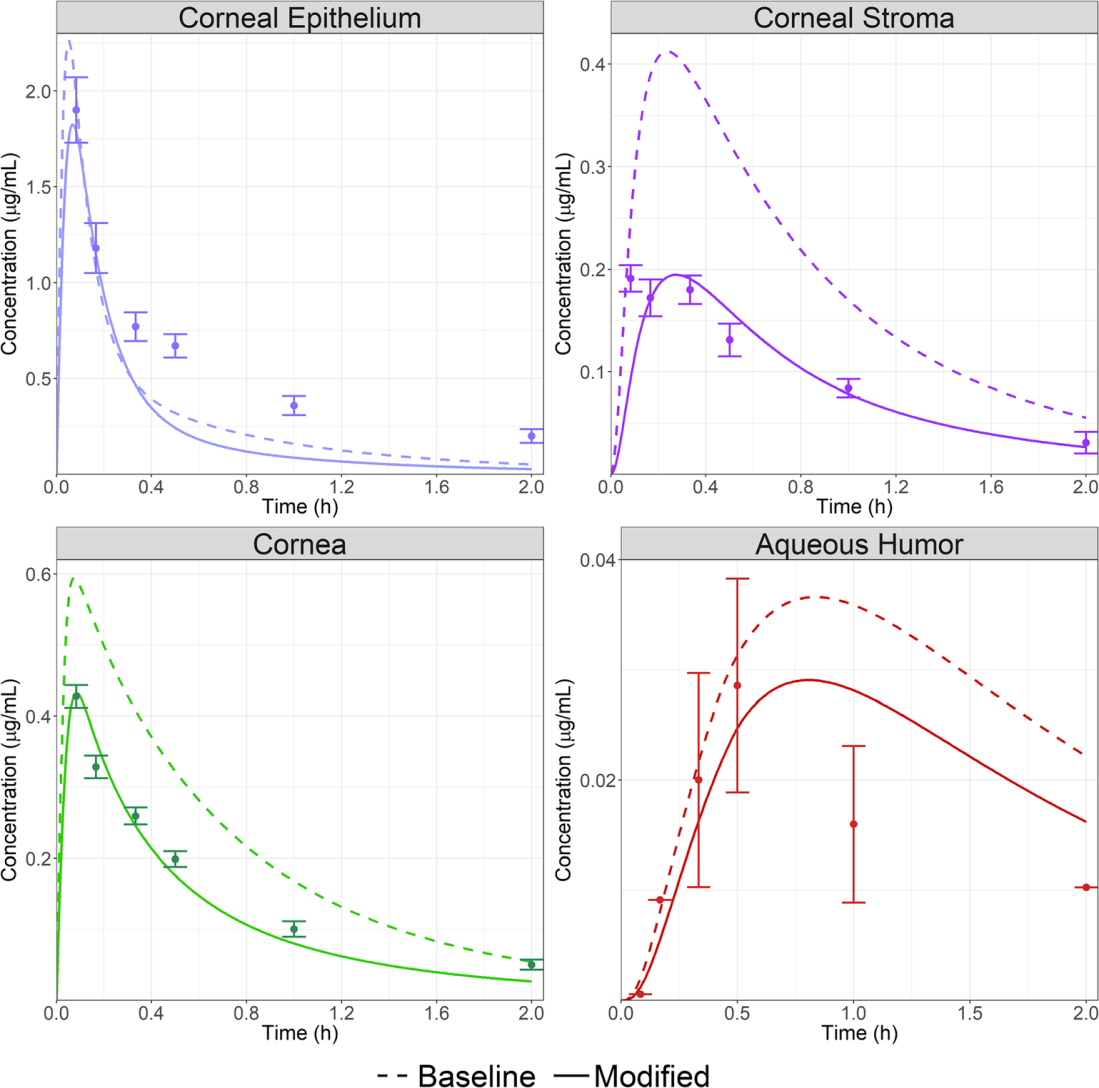
Corneal epithelium (top left), Corneal stroma (top right), Cornea (bottom left) and AH (bottom right) concentrations time course following the administration of 50 μL of Flm solution 4E-5 M to rabbit. Dots represent observed data (30) and lines are model simulations. Dashed lines: model with cornea permeability from in vitro measurement. Solid lines: final model with manual optimization of both cornea (stroma and epithelium) and AH permeabilities.

The initial model was developed against cornea and AH concentration time course data following the administration of 50 μL of a 4E-5 M Flm solution to rabbit (33). Figure 1 (dashed lines) presents the simulation results for the model with all in silico-estimated OCAT™ parameters except for both cornea epithelium and stroma permeabilities that were based on in vitro measurements (32). The final model is shown in Fig. 1 (solid lines) in which corneal epithelium, stroma, and AH permeabilities were optimized to fit the data. This final model reasonably describes cornea epithelium, stroma, and AH concentration time course in rabbit. All parameters used in these simulations are provided in Table I.

**Fig. 2:**
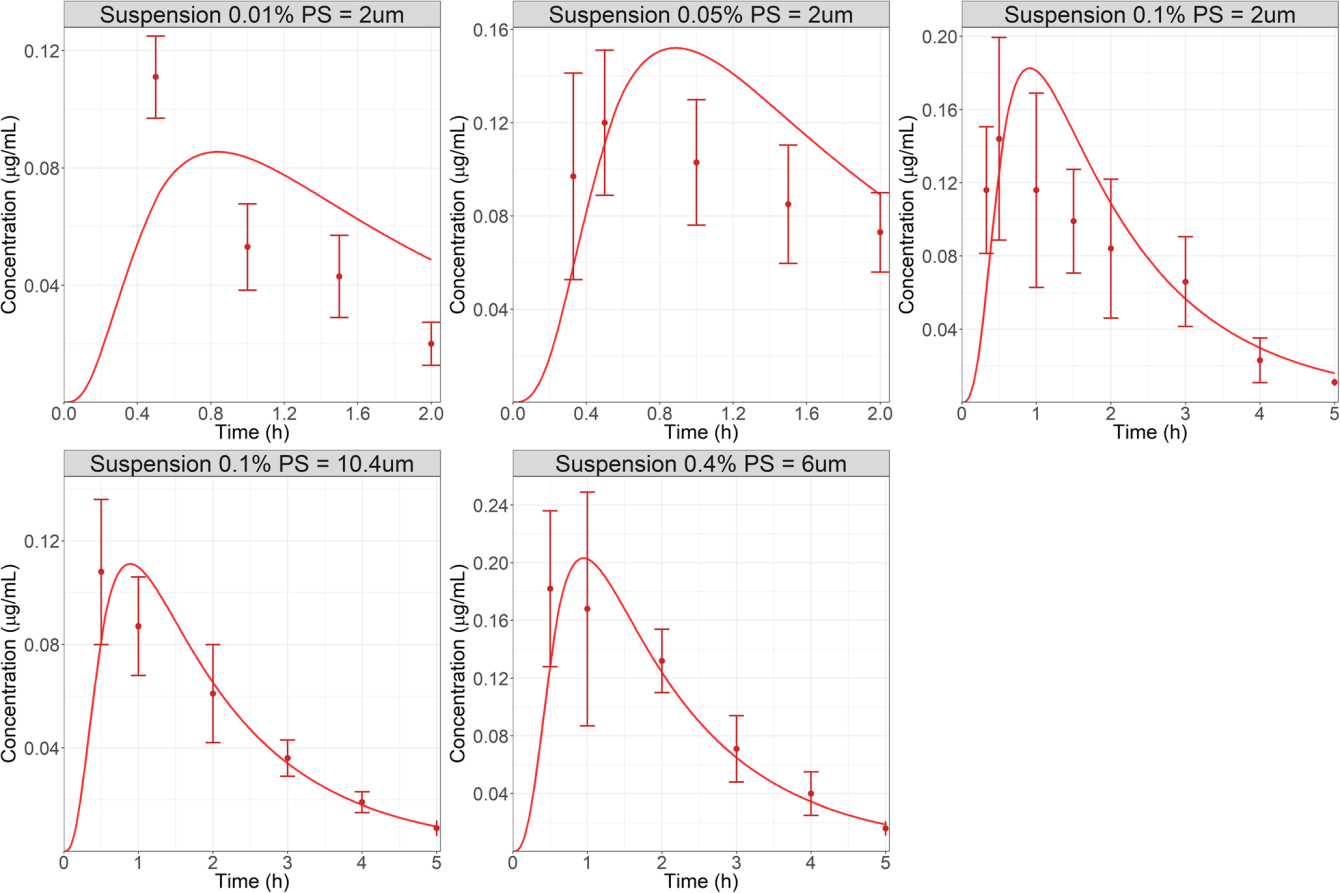
Observed (top row: (31) bottom row: (16)) and simulated AH concentration used in verification steps 1 and 2: administration of three suspensions (PS = 2 μm) with increasing dose (0.01, 0.05 and 0.1%, top row) (31). Administration of two suspensions: 0.1% - PS = 10.4 μm and 0.4% - PS = 6 μm, bottom row (16). Dots represent observed data and lines are model simulations.

The first verification step for this model was to simulate the AH concentration-time course following the administration of three suspensions with different strengths (0.01, 0.05, and 0.1%). These suspensions were modeled using the mixed multiple dose feature of GastroPlus^®^ so that the small amount of dissolved Flm (0.000775 mg) and the remaining mass of suspended particles could be administered at the same time. For these suspensions, the reported mean PS was 2 μm (34). Figure 2 (top row) presents the observed and simulated AH concentration-time course following the administration of three suspensions with increasing dose (0.01, 0.05 and 0.1%). Because the developed OCAT™ model incorporated both nasolacrimal drainage and tear flow clearance mechanisms for solid particles in the pre-cornea compartment, and by factoring in the inter-study variability that is known to be significant in rabbit ocular PK studies, the model successfully predicted the pronounced dose nonlinearity for Flm suspensions observed in AH. Therefore, the first verification step was adequate.

For the second verification step, AH concentration-time courses following the administration of two suspensions of Flm with different doses administered (0.1 and 0.4%) and particle size (mean diameter 6 and 10.4 μm, respectively) were extracted from literature (18). Figure 2 (bottom row) shows the simulations obtained for these two suspensions. The OCAT™ model captured both AH PK profiles, therefore demonstrating the ability to simulate Flm ocular exposure following the administration of multiple formulations with different doses and PS. With both verification steps completed, the model was used to test the new ointment formulation model.

**Fig. 3:**
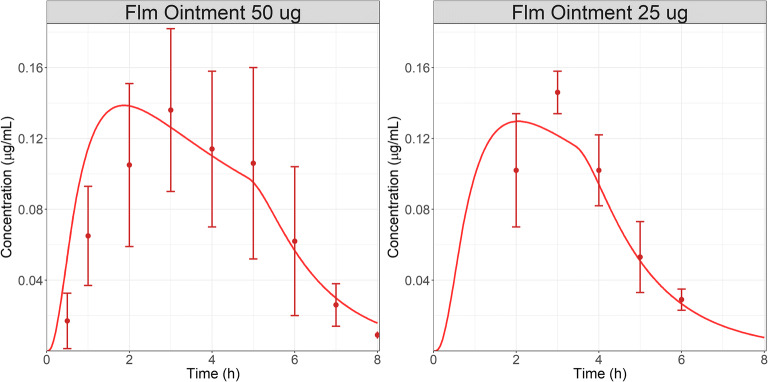
AH concentration (31) following the administration of 50 μg (left) or 25 μg (right) Flm ointment 0.1% to rabbit. Dots represent observed data and lines are model simulations.

AH concentration-time courses following the administration of two similar 0.1% ointments (25 and 50 μL corresponding in two doses: 25 and 50 μg) were extracted from literature (34). Application SA is not described by the authors and it was assumed to be similar to the total cornea SA in both cases. The Higuchi release constant and application time were manually optimized to first describe the data for the 50 μg dose administration. Fig. 3 (left) presents the results for AH concentration following the administration of Flm 0.1% ointment at dose of 50 μg (50 μL) to rabbit. By selecting an application time of 5 h, the model describes the observed data reasonably well. The final optimized Higuchi constant was 2.7E-6 mg/(s^1/2^ cm^2^).

As the Higuchi release constant is related to the ointment structure, the same value was used for the 25 μg dose. The volume administered was expected to have an impact on the overall application time; therefore, this parameter was adjusted for the 25 μg dose. Figure 3 (right) presents the results for AH concentration following the administration of Flm ointment 0.1% at dose of 25 μg (25 μL) to rabbit. The model reasonably described the observed data with an application time of 3.5 h.

Based on this case study, it seems that the developed ointment model described the observed AH data for Flm ointment formulations. Although the information relative to ointment composition are not available, because data following the administration of two doses of the same ointment are published, the following observations can be made. The Higuchi release constant was linked to the ointment formulation and did not vary with the administered dose/volume. However, the application time was dependent on the total volume administered, and it decreased as the volume decreased from 50 to 25 μL. Due to blinking, the administered SA was assumed to be the entire cornea surface.

### Dex Case Study

**Fig. 4:**
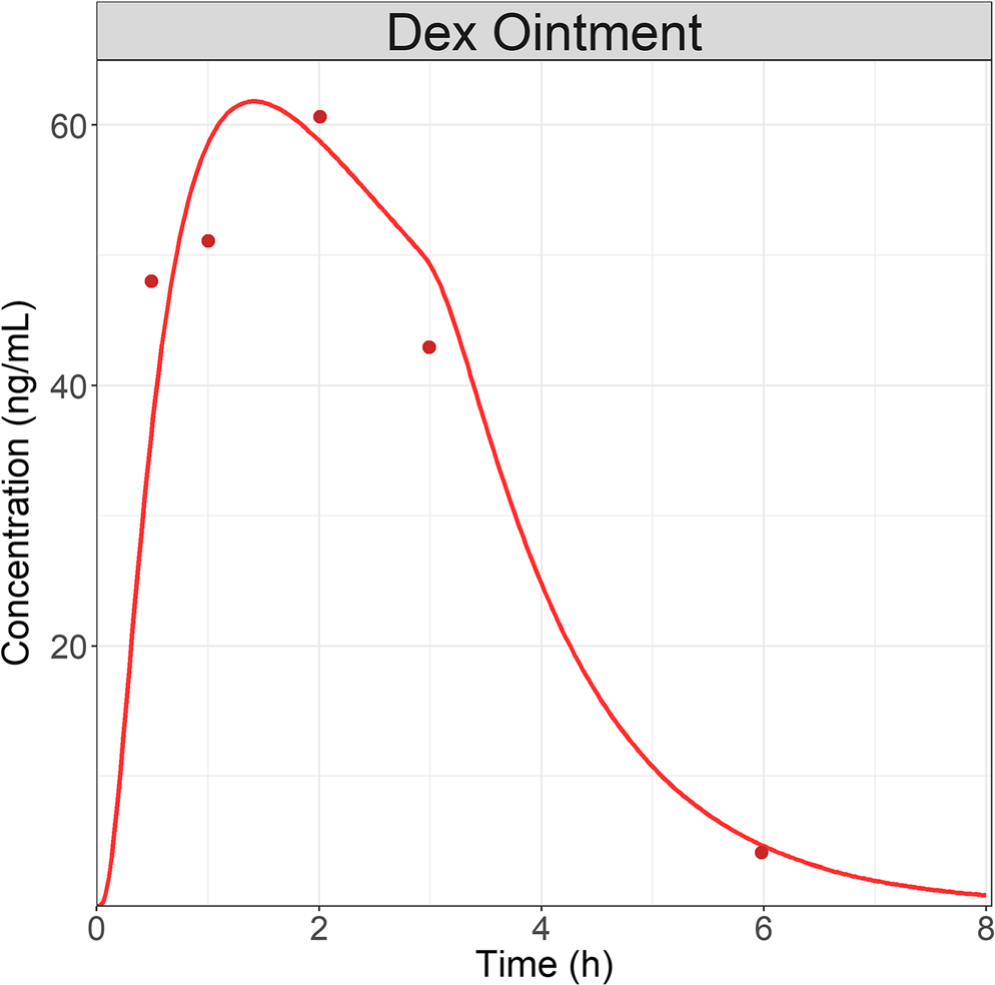
AH concentration following a single administration of Dex 0.1% (30 μg) ointment to rabbit. Dots represent observed AH data and line is the model simulations.

Dex AH concentration following the administration of a 0.1% ointment (30 μL corresponding to 30 μg) were extracted from literature (36). Application SA is not described by the authors and it was assumed to be similar to the total cornea SA. Ointment specific parameters had to be manually optimized to describe the data. A final time of contact of 3.5 h combined with the Higuchi constant of 1.5E-5 mg/(s^1/2^ cm^2^) allowed us to best describe the observed data (Fig. 4). Therefore, we can conclude that an ocular PBPK model, previously validated for solution and suspension formulations (28), can be extended to ointment formulation to describe the ocular concentration following a single administration to the rabbit eye.

### Parameter Sensitivity Analysis

**Fig. 5:**
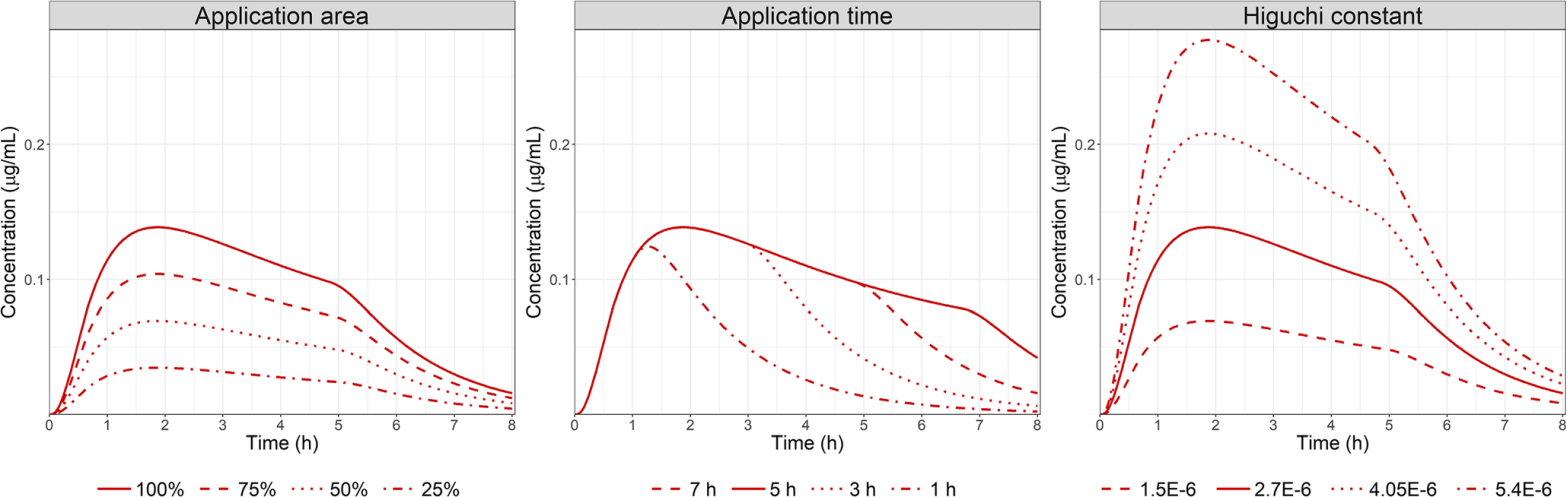
PSA for: Left, application SA assuming the SA is 25, 50, 75 and 100% of the cornea surface; Middle: application time from 1 to 7 h; Right: Higuchi rate constant, following the administration of Flm ointment 0.1% at a dose of 50 μg (50 μL) to rabbit. Solid lines represent the baseline simulations for Flm ointment 0.1% (50 μg) to rabbit presented in Fig. 5.

Using the verified model for Flm ointment in rabbit, a PSA was performed for the three main parameters influencing AH PK dynamic for ointment: application SA, Higuchi rate constant, and application time. Figure 5 presents the AH concentration time course following the administration of Flm ointment 0.1% at a dose of 50 μg (50 μL) to rabbit under the different scenarios. Application SA expressed as a percentage of cornea SA is a critical parameter influencing both Cmax and ocular exposure, but it has a limited impact on Tmax. On the contrary, application time influences all PK metrics: Cmax, AUC, and Tmax. The shorter the application time, the less drug that penetrates the corneal barrier therefore, limiting the time to achieve Cmax and exposure. Interestingly, it seems that after a certain time after dosing (2 h in our PSA for Flm ointment), application time will not influence the absorption phase but only the elimination phase. This indicates that the ointment is acting as a reservoir on the surface of the eye, balancing Flm ocular clearance. Once the ointment is completely washed out of the surface of the eye, Flm has the same AH elimination rate as if it were administered as a solution or suspension. Based on case study 1, it seems the application time is dependent on the total volume administered. This demonstrates a significant influence of the administered volume for ointment formulation on AH exposure. The Higuchi release constant has the most significant impact on both AH Cmax and AUC. Based on Case study 1, the Higuchi rate constant is linked to the ointment formulation. This indicates that formulation characteristics regarding API release rate will be significant to achieve a certain ocular exposure driving the expected effect.

## Discussion

The understanding of ocular absorption mechanism is necessary for both the pharmaceutical industries and the regulatory agencies to support the development and the evaluation of new and generic drug products. Previous work presented the development and validation of an OCAT™-PBPK model by investigating the impact of physiochemical properties of ophthalmic suspensions on in vivo ocular drug absorption and disposition (28). This study done in rabbits was a proof of concept of the benefit of in silico approaches for the development of ophthalmic drug product. This study provides a baseline model capable of simulating the ocular exposure of APIs using both physiological and formulation characteristics. Also, the method served to provide an improved platform, to aid in the development and regulatory assessment of ophthalmic ointment drugs.

Ointments are an important type of semisolid dosage form for the ophthalmic delivery of APIs. These formulations vary in type depending on the choice of bases: oleaginous (hydrocarbon) bases, absorption bases, water-soluble bases, emulsifying bases, etc. APIs may be present inside the ointment in different states: either as a dispersion or as a molecular solution. It is expected, depending on the choice of ointment bases and state of the drug, its release from ointment may vary in both in vitro and in vivo conditions (11). Ophthalmic ointments tend to increase the exposure in ocular tissues compared to solutions/suspensions (37). Therefore, ointment formulations are essentially ophthalmic controlled-release (CR) formulations. For oral CR products, the power of in silico approaches in pharmaceutical research and development as well as regulatory assessment are generally accepted. In 1997, the US FDA published a guidance that includes its recommendations on the use of in silico based in vitro-in vivo correlation (IVIVC) models for extended release oral dosage forms (38). Therefore, in silico methods seem to be a promising approach to support ophthalmic ointment development and approval. However, for ocular products, our understanding of the interaction between formulation and surface of the eye in vivo is not as understood as it is for oral products and the gastrointestinal tract. Despite this limitation, the OCAT™ model proposed here is a necessary first step to identify key mechanisms influencing ocular absorption of APIs administered as ointments. Another limitation this study is facing is small number of case studies available. Indeed our literature search found only two case studies for model validation (34,36), and in these sources, the ointment bases were not defined. Therefore, although our models can simulate the AH concentration time course for both Flm and Dex, the fitted parameters for Higuchi rate constant, application time, and SA may not be transposable to other formulations where the composition is different. Yet, model development processes of these ophthalmic CR formulations are comparable to an oral CR. Indeed, solution and suspensions data were first described to calibrate the model such as intravenous and immediate release data are used for oral CR. This strategy allows one to lock in all absorption, distribution and elimination parameters and then only optimizing the ointment formulation related ones.

In the OCAT™ model, ointment formulation behaves as CR formulation and the transfer of API from the ointment into the tears is described by the Higuchi equation in vivo (E1). PSA analysis demonstrated the Higuchi rate constant is the major parameter influencing the ocular exposure for APIs administered as ointments. However, multiple release models have been identified in vitro for these formulations. The two main ones presented are the transient-boundary layer and Higuchi models (6,11,12). The transient-boundary layer model can describe the in vitro cumulative drug release experiments using a synthetic membrane. On the other hand, the Higuchi model was the best model to describe the in vitro cornea permeation experiments. As in vitro cornea permeation exploration is the closest to in vivo studies by using fresh rabbit cornea tissue, we decided that the Higuchi model was the best approach to describe the API release during in vivo PK studies in rabbit. The ability of our OCAT™ model to capture the AH concentration for both Dex and Flm ointments seems to validate that choice for the release mechanism. However, a publication by Siepmann *et al.* provides an simple equation to estimate the Higuchi constant (SQRT(2*Cini*D*Cs)) where Cini is the initial concentration of the API in the ointment base, D is the reduced diffusion coefficient of the API in the ointment base, and Cs is the solubility of the API in the ointment base (30). Wurster *et al.* measured the diffusion coefficient of several small molecules in an anhydrous lanolin base (39). The diffusion coefficients ranged from pyridine (7.2E-7 cm2/s, MWt = 79.1) to 4-Chloro-4′-fluorobutyrophenone (5.6E-8 cm2/s, MWt = 200.6). Flm has MWt = 376.5 so we might assume the diffusion coefficient is less than 4-Chloro-4′-fluorobutyrophenone. Assuming a corneal SA of 1.76 cm2, and initial concentration of 1 mg/cm3, aqueous solubility, and a diffusion coefficient of 9.6E-9 cm2/s, we can calculate the k_Higuchi_ to be 1.73E-5 mg/(s1/2 cm2). However, that value is ~6.5 times greater than the fitted k_Higuchi_ (2.7E-6 mg/(s1/2 cm2)). But the diffusion coefficient estimated in literature are for liquid compounds and it is known that Flm is present in solid state within the ointment base. This could decrease Flm diffusion coefficient. Also, it is expected the APIs would diffuse more freely in an anhydrous lanolin base as described by Wurster *et al.,* *versus* a petroleum base typically used in ointment formulations. Reducing the diffusion by two orders of magnitude helps to reconcile the calculated and fitted k_Higuchi_. To test the diffusion coefficient parameter that is extracted from literature, it was used for acyclovir ointment. The calculated k_Higuchi_ for a 6% dose (Cs = 1.4 mg/mL, assumed diffusion coefficient: 5.6E-8 cm2/s) is around 200 greater than the measured value in vitro (6). As the exact composition of Flm ointments is unknown, it is not possible to explore a relationship between the ointment characteristics and the expected in vitro release. However, the acyclovir in vitro data seems to suggest the diffusion coefficients obtained from several small molecules in an anhydrous lanolin base (39) are not representative of an ocular ointment used either in vitro or in vivo. Specific measurement of this parameter may be required to predict the Higuchi release constant. As more experience is gained with new case studies using well known formulations, explorations based on calculation with accurate diffusion coefficients or in vitro measurements should be feasible in the future.

The PSA for application/absorption SA demonstrates a significant impact on absorption rate in the AH. Although the OCAT™ model captured the AH concentration time course for Flm, it seems the absorption phase is slightly overpredicted compared to the observed data for both administrations (Fig. 3: 25 and 50 μL). Our current model assumes the absorption surface area to be constant with time and equivalent to the cornea SA. Although, blinking is expected to disperse the formulation across the cornea, the limited blinking rate observed in rabbit (every twenty minutes) (19) may provide enough time to the formulation to settle in the conjunctiva sac; therefore, reducing the ocular absorption by limiting the absorption surface area. A dynamic application/absorption SA linked to the blinking rate may enhance our prediction of the Flm ointments. However, for human the formulation settling may not happen as the blinking rate (19) is higher and a constant absorption SA may be a reasonable assumption.

For Flm and Dex, ophthalmic ointments result in a longer ocular exposure in AH compared to solution and suspensions. Indeed, for Flm, the AH AUC is increased by 100% when the drug is administered at the same dose but using an ointment vehicle *versus* a suspension. The ointment will influence the residence time as the ointment base present at the surface of the eye can act as a reservoir for drug release in ocular tissues. Nevertheless, despite the improved exposure, most of the drug does not permeate in the ocular tissues and is eliminated from the surface of the eye, such as for suspensions and solutions. This elimination mechanism has not been clearly identified and may be linked to dynamic forces mediated by both the blinking process and the tears drainage. In the OCAT™ model, application time defines a very slow zero-order elimination driving the transfer of the unreleased fraction of the API through the nasolacrimal duct directly into the stomach. This parameter was necessary to capture the elimination part of the observed AH PK profiles that seems to happen in two phases. In the first phase the elimination is balanced by the presence of API in the ointment acting as a reservoir. Then in the second phase, once all the material had been removed from the ocular surface, the elimination rate is similar to the one simulated for solution and suspension. This seems to be confirmed by the Flm case study. Indeed, the 0.1% formulation was administered with two different volumes: 25 and 50 μL. To capture the AH PK profiles, the application time for the lowest volume is significantly different than the one obtained for the higher volume, demonstrating a clear impact of the administered volume on the residence time of the formulation on the ocular surface and therefore, the ocular exposure. As the elimination mechanism of the ointment base and the entrapped material is better understood, this process may need to be revised in future iterations of the OCAT™ model. However, it is not a limiting factor to capture the observed concentration, as well as investigating formulation changes impact on ocular exposure.

In conclusion, we have successfully developed and verified an OCAT™ model for APIs administered as ointment formulations. We have been able to describe the AH concentration course for both Flm and Dex ocular ointments in rabbits. Based on our model simulations, the critical parameters influencing ocular PK metrics are application time, administration SA, and API release rate. Ideally, the Higuchi rate constant could be further broken down into various formulation factors, measurable in vitro, such as PS distribution, ointment base type and rheological characteristics. So far, the parameters included in the model should capture most of the formulation variants (e.g., Higuchi rate constant should capture the PS distribution and ointment base type, while application time should capture the rheological differences). This tool may support drug development and provide a better understanding of the impact of formulation modifications on the in vivo performance of ophthalmic ointments products. A deeper understanding of key physiological mechanisms influencing PK outcomes as well as the extrapolation from rabbit to human model are the next steps planned for this OCAT™ ointment model.

## Supplementary Information

ESM 1 (DOCX 84 kb)
