# Supplementary material for: Ocular Physiologically Based Pharmacokinetic Modeling for Ointment Formulations
Source: Pharm Res. 2020 Nov 19;37(12):245. doi: 10.1007/s11095-020-02965-y (PMC7677276; doi:10.1007/s11095-020-02965-y)
Supplement: Supplementary file 1 — (DOCX 84 kb) [file 11095_2020_2965_MOESM1_ESM.docx]

#### Ocular Compartmental Absorption & Transit Model (OCAT)

The ocular drug delivery model can simulate a variety of ocular dosage forms, including both the front of the eye (topical solution and suspension eye drops) and the back (intravitreal injections) as immediate release ocular dosage forms, as well as controlled release dosage forms that simulate drug delivery via intravitrealintra-vitreal or subconjunctival implants. The OCAT model represents the eye as a collection of the following compartments: pre-cornea, corneal epithelium, corneal stroma, bulbar conjunctiva, palpebral conjunctiva, aqueous humor, anterior sclera, posterior sclera, iris-ciliary body, choroid-RPE (a combination of choroid and the retinal pigment epithelium), retina, anterior and posterior vitreous humor. The lens is not considered as an additional compartment but serves as a barrier separating the aqueous humor and anterior vitreous humor compartments. The sclera and vitreous humor have been split to account for differences in mass transfer characteristics (convective flow vs no convective flow). The OCAT model as implemented in GastroPlus™ version 9.6 has been parameterized with physiological system characteristics for human, rabbit, and cynomolgus monkey eyes. A schematic diagram showing how the different compartments are connected to one another and the rest of the body is shown in Figure 1.


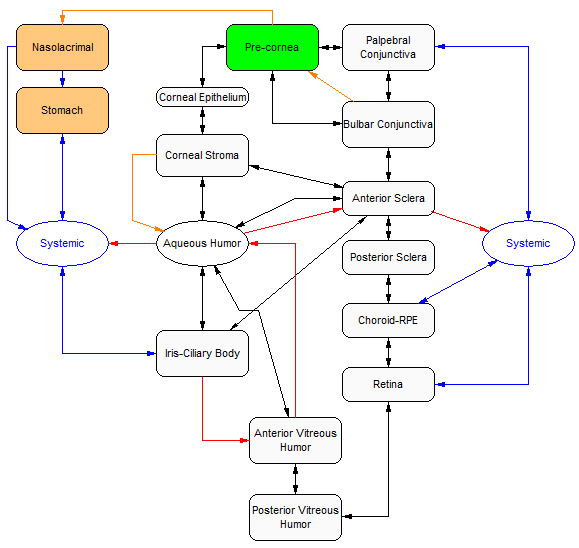


##### Figure 1. OCAT model schematic

Black arrows represent the drug exchange between adjacent ocular compartments via passive diffusion and/or carrier mediated transport, orange and red arrows represent the drug movement due to convective fluid flow between compartments, blue arrows represent the drug exchange between perfused ocular tissues and systemic circulation via passive diffusion and/or carrier-mediated transport.

Once the drug is dosed in a user selected ocular compartment, it is subjected to a variety of mechanisms resulting in drug loss, productive absorption, distribution and clearance. For topical doses (eye drops) dosed entirely into the pre-cornea, the base volume of the pre-cornea compartment (tear film and conjunctival sac) is temporarily increased in order to instill a volume of the drug formulation but cannot exceed the maximal precorneal volume for a given species. This volume dynamically increases due to tear production and then gradually returns to the base volume. Excess fluid (in excess of the maximal volume) is automatically pushed out of the eye by blinking and dissolved and suspended drug is lost for productive ocular or non-productive oral absorption. The remaining volume of a solution or a combination of dissolved and suspended drug can be transported from the pre-corneal compartment to the nasolacrimal duct. Fresh tears can also wash the drug away from the pre-cornea into the nasolacrimal duct. Collectively, both the gradual volume reduction and tear flow constitute nasolacrimal drainage and the model assumes these to be first–order processes, given by the following equation.

$NasolacrimalRate=DRC\times C_{PCA}\times\left(V_{PCA}\left(t\right)-V_{PCA}^{phys}\right)+TFR\times C_{PCA}$ Equation (1)

where *DRC* and *TFR* are first order drainage rate constant (1/min) and the constant tear flow rate (μL/min, respectively), $C_{PCA}$ is the pre-corneal drug concentration, $V_{PCA}\left(t\right)$ is the instantaneous pre-corneal volume, and $V_{PCA}^{phys}$ is the base physiological volume of the pre-cornea. A large portion of the drug entering the nasolacrimal duct gets absorbed directly into systemic circulation, while the remainder is swallowed and reaches the stomach. A user-defined fraction of drug is transferred from the nasolacrimal duct directly into systemic circulation ($f_{Nasolac}^{Sys}$ ) given as:

$\begin{matrix} NasolacrimalSysRate=f_{Nasolac}^{Sys}\times NasolacrimalRate \\ NasolacrimalStomachRate=\left(1-f_{Nasolac}^{Sys}\right)\times NasolacrimalRate \end{matrix}$ Equation (2)

When the formulation is a suspension, undissolved drug that drains into the nasolacrimal duct is assumed to enter the stomach.

**Transport into adjacent ocular compartment(s):** The mass of drug moves from one compartment into adjacent compartments via a combination of passive diffusion ($AbsRatePass$) and carrier mediated transport ($AbsRateCar$).

$AbsRate_{A\to B}=AbsRatePass_{A\to B}+AbsRateCar_{A\to B}$ Equation (3)

Simple diffusion is modeled using a mechanistic (permeability-surface area) approach (Equations 4) The relevant equations for drug diffusion between any two compartments marked *A* and *B* are given as:

**Passive Diffusion: Permeability-Surface Area:**

$\begin{matrix} AbsRatePass_{A\to B}=P_{B}\times AbsSA_{A-B}\times\left(C_{A}-C_{B}\right) \\ AbsRatePass_{B\to A}=P_{A}\times AbsSA_{A-B}\times\left(C_{B}-C_{A}\right) \end{matrix}$ Equation (4)

In Equation 4, *P_x_* represents permeability, *AbsSA* represents contact surface area between compartments that is used for diffusion, and *C* represents unbound concentration. The subscripts represent the relevant compartments. It should be noted that in moving from compartment A to B the permeability assigned to compartment B is used to calculate the rate coefficient, but a positive concentration gradient term (*C_A_-C_B_*) would be related to mass movement down the concentration gradient from A to B.

**Carrier-mediated Transport:**

$\frac{{dM}_{A}}{dt} =\sum_{i=1}^{N_{B\to A}^{trans}} \left( \frac{Expr_{i}\times V_{max, i}\times C_{B}}{K_{m,i}+C_{B}} \right)-\sum_{i=1}^{N_{A\to B}^{trans}} \left( \frac{Expr_{i}\times V_{max, i}\times C_{A}}{K_{m,i}+C_{A}} \right)$ Equation (5)

In Equation 5 the transporter active site is defined for compartment (A) and the mass moves to the receiver compartment (B). Also, $N_{A\to B}^{trans}$ and $N_{B\to A}^{trans}$, respectively, represent the number of transporters along the relevant direction, *Expr* denotes the relative or absolute protein expression for the transporter, *V_max_* and *K_m_* represent parameters for saturable transport, and *C* represents unbound concentration.

**Metabolism:** This can be described as either a linear or a saturable process, through the following mathematical expressions:

**Linear:**

$MetabRate\left( {mg}/s \right)={CL}_{metab}\times C$ Equation (6)

**Saturable:**

$MetabRate=\sum_{i=1}^{N_{enz}} \left(\frac{Expr_{i}\times V_{\max,i}\times C}{K_{m,i}+C}\right)$ Equation (7)

In Equations 6 and 7, *CL_metab_* represents a fixed (linear) intrinsic clearance in L/h, *V* is volume of given ocular compartment, ***V_max_*** and *K_m_* represent Michaelis-Menten saturable enzymatic parameters, *C* represents unbound drug concentration and *Expr* denotes expression of enzymes.

**Systemic Absorption:** Relevant only for perfused ocular compartments (palpebral conjunctiva, iris-ciliary body, chroroid-RPE and the retina). It is a combination of a first-order (passive) process and carrier-mediated process as:

$CompToSCRate=k_{i}^{sys}\times V\times\left(C-C_{sys}\right)+\sum_{i=1}^{N_{trans}^{eff}} \left(\frac{Expr_{i}\times V_{\max,i}\times C}{K_{m,i}+C}\right)-\sum_{i=1}^{N_{trans}^{\inf}} \left(\frac{Expr_{i}\times V_{\max,i}\times C_{sys}}{K_{m,i}+C_{sys}}\right)$ Equation (8)

In equation 8, $k_{i}^{sys}$ is the systemic absorption rate constant for given compartment, *V* is the volume of the compartment, *C* is the unbound drug concentration in ocular compartment, $C_{sys}$ is the unbound drug concentration in plasma, $N_{trans}^{\text{inf}}$ and $N_{trans}^{eff}$ represent the number of influx and efflux transporters, respectively, between a compartment and systemic circulation, *Expr* denotes expression of given transporter in an ocular compartment. In addition to these systemic absorption processes, the OCAT model includes mass flow by convective pathways from aqueous humor and anterior sclera to the systemic circulation.

**Melanin Binding:** This represents reversible binding of the drug to melanin in certain compartments of the eye. These compartments are the iris-ciliary body and choroid-RPE. It is described as either a linear or a saturable process, through the following mathematical expressions:

**Linear:**

$m_{free}=f_{melanin}^{unbound}\times m_{tot}$ Equation (9)

**Saturable:**

$m_{tot}=m_{free}\times\left( 1+\frac{b_{1}}{a_{1}+m_{free}}+\frac{b_{2}}{a_{2}+m_{free}} \right)$ Equation (10)

In Equations 9 and 10, $m_{free}$ and $m_{tot}$, respectively, represent the free (unbound) and total amount of the drug in a given compartment. $f_{melanin}^{unbound}$, in a given compartment, is the fixed fraction of the drug that is not bound to melanin at any given time (similar to fixed fraction unbound in plasma or *fup*). In Equation 10, $b_{1}$, $b_{2}$, $a_{1}$ and $a_{2}$ are given as:

$b_{1}=M_{melanin}\times B_{\max}\times10^{-6}\times MW$ Equation (11)

$b_{2}=M_{melanin}\times B_{\max,slow}\times10^{-6}\times MW$ Equation (12)

$a_{1}=K_{d}\times V\times10^{-6}\times MW$ Equation (13)

$a_{2}=K_{d,slow}\times V\times10^{-6}\times MW$ Equation (14)

In equations 11 through 14, *M_melanin_* is the amount of melanin in given compartment, *MW* is the molecular weight of the drug, and *V* s the volume of the given compartment. *B_max_* and *B_max,slow_* (in μmol/g) and *K_d_* and *K_d,slow_* ($\mu M$) represent drug-dependent equilibrium Langmuir saturable binding dissociation constants (accounting for two different binding sites).

*Please note that Equation 10 leads to the free amount being calculated from the total amount through reverse functional mapping using an iterative Newton’s method within the program*
